# Quantity and quality of seed dispersal by a large arboreal frugivore in small and large Atlantic forest fragments

**DOI:** 10.1371/journal.pone.0193660

**Published:** 2018-03-21

**Authors:** Óscar M. Chaves, Júlio César Bicca-Marques, Colin A. Chapman

**Affiliations:** 1 Pontifícia Universidade Católica do Rio Grande do Sul, Escola de Ciências, Porto Alegre, Rio Grande do Sul, Brazil; 2 Organização Fauna Brasilis, Rua Manuel Vieira da Rosa No. 108, Lami, Porto Alegre, Rio Grande do Sul, Brazil; 3 McGill School of Environment and Department of Anthropology, McGill University, Montreal, Quebec, Canada; Sichuan University, CHINA

## Abstract

Seed dispersal is a key process driving the structure, composition, and regeneration of tropical forests. Larger frugivores play a crucial role in community structuring by dispersing large seeds not dispersed by smaller frugivores. We assessed the hypothesis that brown howler monkeys (*Alouatta guariba clamitans*) provide seed dispersal services for a wide assemblage of plant species in both small and large Atlantic forest fragments. Although fruit availability often decreases in small fragments compared with large ones, we predicted that brown howlers are efficient seed dispersers in quantitative and qualitative terms in both forest types given their high dietary flexibility. After a 36-month study period and 2,962 sampling hours, we found that howlers swallowed and defecated intact the vast majority of seeds (96%-100%) they handled in all study sites. Overall, they defecated ca. 315,600 seeds belonging to 98 species distributed in eight growth forms. We estimated that each individual howler dispersed an average of 143 (SD = 49) seeds >2 mm per day or 52,052 (SD = 17,782) seeds per year. They dispersed seeds of 58% to 93% of the local assemblages of fleshy-fruit trees. In most cases, the richness and abundance of seed species dispersed was similar between small and large fragments. However, groups inhabiting small fragments tended to disperse a higher diversity of seeds from rarely consumed fruits than those living in large fragments. We conclude that brown howlers are legitimate seed dispersers for most fleshy-fruit species of the angiosperm assemblages of their habitats, and that they might favor the regeneration of Atlantic forest fragments with the plentiful amount of intact seeds that they disperse each year.

## Introduction

Seed dispersal is a crucial ecological process that influences the local persistence and fitness of flowering species and that modulates the composition and diversity of plant communities [1–3]. The dispersal reduces density-dependent and distance-dependent seed and seedling mortality while favoring seed germination, gene flow, and the colonization of new habitats [2, 4–6]. Overall, quantitative (e.g. tree visitation frequency, number and richness of dispersed seeds) and qualitative (e.g. seed handling and damage level of defecated seeds) factors influence the effectiveness of a seed disperser [1, 6, 7].

Vertebrates disperse seeds of 70% to 90% of woody plants of tropical forests [8, 9], playing a critical role in their regeneration [3, 10, 11]. Primates represent 25% to 40% of the frugivore biomass in these ecosystems [12, 13]. The service these animals provide in primary seed dispersal and seedling recruitment is particularly relevant for the regeneration of fragmented forest ecosystems inhabited by impoverished guilds of frugivores [e.g. 12, 14–20], such as the Atlantic forest [10, 18], a biodiversity hotspot severely affected by an ongoing process of deforestation, defaunation, urbanization, and other unsustainable human activities [10, 21, 22]. This biome is split in ~245,000 forest fragments highly variable in size, form, and degree of isolation that together cover <12% of its original extension [21]. The Atlantic forest is habitat to 24 primate species, 18 of which are endemic [23], whose contribution to forest regeneration and restoration via seed dispersal remains poorly studied [18].

Primate diversity in the southern, subtropical portion of the Atlantic forest is much lower. Here, the brown howler monkey (*Alouatta guariba clamitans*) is the largest, more abundant and, sometimes, unique primate. Like its congeners [see 24, 25], brown howlers cope well with a reduction in the availability of wild foods in small forest fragments [26, 27]. They are folivorous-frugivorous consumers that invest heavily in fruit feeding whenever possible [26–28]. This frugivory opportunism qualifies them as potentially important seed dispersers of many Atlantic forest plant species, especially in habitats lacking other large frugivores [10, 18, 29]. For instance, Atlantic forest *Alouatta* species disperse seeds of a minimum of 74 plant species that represent >90% of the species that they handle. This service qualifies them as the third most important primate seed disperser of the Atlantic forest, just behind *Sapajus* spp. and *Leontopithecus* spp. [18].

However, it is unclear whether the efficiency of primates as seed dispersers is constrained in small and/or highly disturbed Atlantic forest fragments characterized by lower wild fruit availablity [24, 30–32]. The richness of dispersed seeds can be lower and the proportion of predated or dropped seeds under parent trees can be higher in these environments, as observed in atelids from other biomes (e.g. *Alouatta seniculus* [33], *Ateles geoffroyi* [17]). Nevertheless, the capacity of brown howlers to complement their diets with second-choice wild fruits or cultivated fruits [26–28] could compensate this potential decrease of preferred wild fruits in those fragments.

We tested the hypothesis that brown howlers provide highly efficient quantitative and qualitative seed dispersal services for a wide assemblage of plant species in both small and large Atlantic forest remnants. Specifically, we compared the quality of seed dispersal services provided by brown howlers in small and large Atlantic forest fragments during a 36-month period in terms of (i) the richness and abundance of dispersed seeds at the level of forest (i.e. each study fragment) and fecal sample, (ii) the proportion of the tree species assemblage that is dispersed in each fragment (an analysis so far neglected in previous studies), (iii) the alpha-diversity of rare, common, and dominant dispersed seeds in fecal samples, (iv) the types of seed handling applied, and (v) the proportion of seeds that are defecated intact. We predicted that these animals are similarly efficient seed dispersers in both forest types, despite the potential decrease in wild fruit availability in small fragments.

## Materials and methods

### Study fragments and monkey groups

We studied six howler monkey groups (6–10 individuals each) inhabiting three small (<10 ha) fragments S1 (30°11'00".1 S, 51°06'06.6" W), S2 (30°12'18.4" S, 51°06'05.7" W), and S3 (30°12'26.6" S, 51°05'54.0" W) and three large fragments (> 90 ha) L1 (30°23'15.6" S, 51°02'43.3" W), L2 (30°20'56.8" S, 51°02'58.2" W), and L3 (30°10'39.5" S, 51°06'18.2" W) in Porto Alegre and Viamão, Rio Grande do Sul State, southern Brazil. Additional details of group composition and fragment characteristics are available elsewhere [27, 28]. Permissions to work in the study sites were provided by the small fragment's landowners or by governmental authorities of the Secretaria do Meio Ambiente do Rio Grande do Sul (SEMA) and Instituto Chico Mendes de Conservação da Biodiversidade (ICMBio) in the case of the large fragments.

### Behavioral data

We collected data on the feeding ecology of brown howlers during 2,962 observation hours distributed in 291 sampling days over 36 months in four sampling years (June 2011 to June 2014; Table A in S1 Appendix). We monitored each study group from dawn to dusk during four to five consecutive days on a bimonthly basis (total sampling effort per group = 429–550 h, S1 Table in S1 Appendix). We used the instantaneous scan sampling method [34] with 5-min sampling units at 15-min intervals. For this we used high-resolution binoculars (Swarovski® SLC 10 x 42). When adults, sub-adults, and juveniles were feeding on fruit, we recorded the fruit species (or morphospecies), its growth form, and the type of seed handling (i.e. swallowed, chewed, spat out, or dropped under the parent tree [17, 35]). Because this work was a purely observational study, no physical contact with the animals occurred during the study period and all sampling procedures were approved by Animal Care and Use Committee of the Pontifical Catholic University of Rio Grande do Sul as part of obtaining the field permit.

### Fecal sampling and seed identification

We collected fecal samples immediately after defecation. Defecation occurred, on average, four times per day (SD = 0.3, range = 2–8, n = 200 complete days), mainly during or after the main resting periods of early morning (5:30–8:00), midday-early afternoon (11:30–14:00) and late afternoon (16:30–18:00). We stored samples individually in labeled plastic bags. We rinsed individual samples using water and two sieves with decreasing mesh size (4 mm and 0.7 mm).

Seed size of fleshy fruit species ranged from <1 mm (e.g. *Rhipsalis* spp.) to >20 mm (e.g. *Garcinia gardneriana*) in the study region, as tree species with larger seeds are rare in southern Brazil [36]. We identified the seeds at the species or morphospecies level based on a reference collection assembled for this study by ÓMC (available from: http://dx.doi.org/10.17632/ckddcjtrgg.1) and counted the number of seeds of each species in each sample. We estimated the contribution of each species as the proportion of the number of its seeds relative to the total number of seeds from all species multiplied by 100. We considered as top dispersed species those seed species that together contributed with ~80% of total fecal records.

We assessed the damage level of the seed testa using a 14x triplet loupe or a stereomicroscope at 25x magnification. We classified damage into four categories according to the percentage of damage: intact (≤5%), moderate (>5% to ≤25%), medium (>25% to 50%), and heavy (>50%). We analyzed all seeds >2 mm in length. Complementarily, we estimated the damage of up to one hundred 1-mm to 2-mm seeds, such as *Ficus* spp., per fecal sample. In total, we estimated the damage level of 92,243 seeds (full database on the dispersed seed species is available at https://data.mendeley.com/datasets/dm592xxmmn/4).

### Tree species richness

We used our database on tree composition of each fragment to estimate their species richness (range = 48–61, see [27] for full details on tree surveys). We classified tree species according to two major dispersal syndromes: vertebrate-dispersed seeds (species producing diaspora attached to fleshy pulp or aril that monkeys, birds, and other vertebrates eat) and abiotic-dispersed seeds (species producing dry winged seeds or fruits, Table B in S1 Appendix).

### Statistical analysis

#### Richness and abundance of dispersed seed species

We used the mean of three nonparametric richness estimators (Chao1-bc, ACE, and Jack1) calculated over the function ‘ChaoSpecies’ in the R package SpadeR v0.1.0 [37] to determine the expected number of seed species dispersed by each study group. We assessed the sample completeness for each group by dividing the observed number of species by its expected number. We assumed that our sampling effort was sufficient because sample completeness was >79% for most fragments (Table 1). We computed sample-based rarefaction curves to compare the richness of dispersed seeds among brown howler groups using 95% confidence intervals of the moment-based estimator. We used the sample extrapolation procedure [38] to perform these comparisons based on a standardized randomly selected subsample of 225 fecal samples per monkey group. We used a Chi-square test of goodness-of-fit to compare the total abundance of seeds dispersed by brown howler groups during the study period. Furthermore, we used rank-abundance graphs to visualize the relative abundance/dominance of each seed species in the seed dispersed assemblage [39].

**Table 1:** Observed and expected richness of seed species dispersed by brown howlers in Atlantic forest fragments in southern Brazil.

Fragment	S_obs_	Nonparametric richness estimator				
		Chao1-bc	ACE	Jack1	Mean	SC (%)
S1	53 (39, 28)	56.5	58.7	61.0	58.7 (2.3)	90.2
S2	53 (37, 30)	58.1	62.7	62.0	60.5 (2.5)	87.0
S3	30 (23, 14)	35.0	42.8	36.0	37.9 (4.2)	79.1
L1	39 (30, 24)	42.0	45.2	45.0	44.1 (1.8)	88.5
L2	40 (33, 23)	67.5	73.2	51.0	63.9 (11.5)	62.6
L3	43 (35, 26)	45.5	46.5	48.0	46.7 (1.3)	92.1

Observed species richness (S_obs_) and the number of genera and families are indicated in parentheses. The three richness estimators were: bias corrected form for the Chao1 (Chao1-bc), Abundance-based Coverage Estimator (ACE), and 1^st^ order Jackknife (Jack1) that uses the number of singletons to estimate the number of undetected species. The mean (±SD) of the three richness estimators is also indicated. The sample completeness (SC) was calculated as the percentage of expected richness (i.e. observed/mean of estimator x 100) covered by sampling effort.

Finally, we used generalized linear models (GLMs) with Poisson error distribution and log link-function as recommended for count dependent variables [40] to compare the total seed richness (or abundance of seeds >2 mm) per fecal sample among fragments. We identified differences between groups using post-hoc contrasts with the function ‘glht’ of the R package multcomp [41].

#### Alpha-diversity of dispersed seeds

We used multiplicative decompositions of Hill numbers [42, 43] calculated over the function ‘Diversity’ in the R package SpadeR v0.1.0 [37] to estimate the alpha-diversity of dispersed seeds in each fragment. It is possible to determine the Hill numbers at three diversity orders named q orders 0, 1, and 2 [37]. Diversity order 0 represents the diversity of all species without overemphasizing their abundances (i.e. an equivalent of species richness). This order gives a disproportionate weight to ‘rare’ species (i.e. species whose seeds were rarely found in fecal samples). Order 1 is the exponential of Shannon’s entropy. It represents the diversity of ‘common’ species (i.e. species whose seeds were frequently found in fecal samples) because it weights each species according to its abundance without overemphasizing rare or highly abundant species. Finally, order 2 is the inverse of Simpson’s index. It represents the diversity of ‘dominant’ species (i.e. species whose seeds dominated the set of seeds of the fecal samples) in the community by emphasizing abundant species while ignoring rare ones [37, 42].

Hill numbers are expressed in units of ‘species’ and can be plotted on the same graph (named diversity profiles). These profiles provide useful information on the patterns of species abundance and diversity of local assemblages because they allow a fine analysis of the relative importance of rare, common, and dominant species [37]. Detailed information on Hill numbers and mathematical equations for each diversity *q* order are available elsewhere [37, 42, 43]. We used 95% confidence intervals with the bootstrap procedure to compare the diversity orders between small and large fragments. Non-overlapping confidence intervals indicate statistically significant differences.

#### Seed handling and proportion of seeds defecated intact

We used the Chi-square test of goodness-of-fit followed by Bonferroni correction over the function ‘p.adjust’ to compare the number of records in each handling category among groups because we performed multiple comparisons between the same data sets. We report one-tailed p*-*values. Finally, we used GLMs with a quasibinomial error distribution and logit link-function [40] to compare the proportion of seeds that are defecated intact among fragments. We identified pairwise differences between study groups using post-hoc contrasts over the function ‘glht’ of the R package multcomp. We performed all statistical analyses in R v.3.3.2 [44].

### Ethics statement

This study complied with protocols and sampling procedures approved by the Scientific Committee of the Faculty of Biosciences and Animal Care and Use Committee of the Pontifical Catholic University of Rio Grande do Sul (project #3477-SIPESQ) as part of obtaining the field permit. It meets all Brazilian animal care policies and has the required permissions to work in the study Atlantic forest fragments (permits #28578-SISBIO/ICMBio and #372-SEMA, permits of small fragment's landowners Felipe Vianna, Edalina Fernandes, and Eduvaldo Vianna). Because this work was a purely observational study, no physical contact with the animals occurred during the study period. The study also meets all ethical and legal requirements established by the American Society of Primatologists, Animal Care and Use Committee, and the Ethical Committee of the Zoological Society of London for research with nonhuman primates.

## Results

### Richness and abundance of seed species at the forest and fecal sample levels

**Fig 1:**
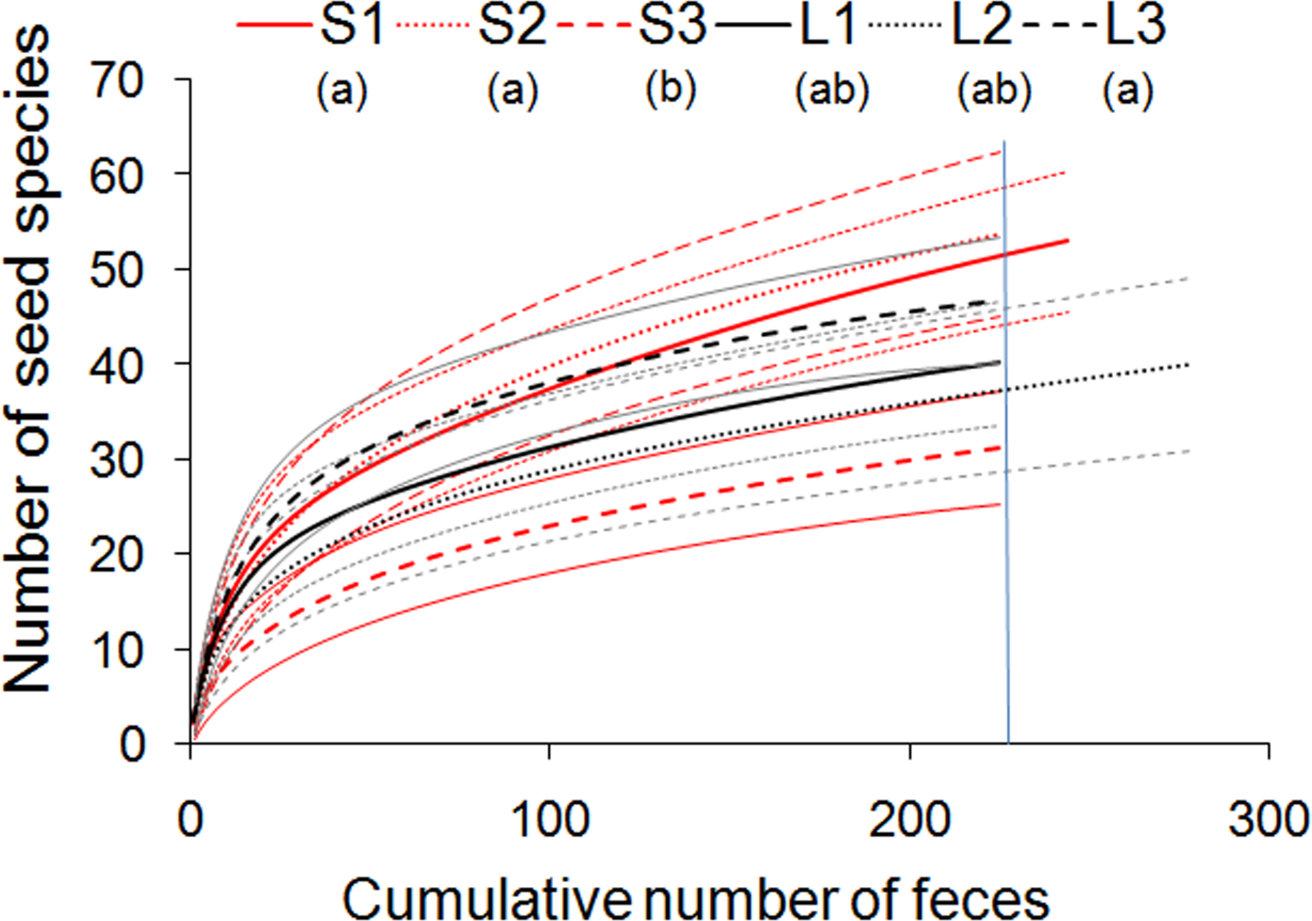
Sample-based rarefaction curves of the seed species richness dispersed by brown howlers in three small (thick red lines) and three large (thick black lines) Atlantic forest fragments. Dashed and fine lines show 95% confidence intervals. Blue vertical line shows the species richness for the rarified number of fecal samples (n = 225). We used 95% confidence intervals of the moment-based estimator to compare the richness of dispersed seeds among groups [38]. Different lower case letters in parentheses indicate significant between-group differences.

Brown howlers defecated ca. 315,600 seeds ranging from <1 mm to 23 mm in length (n = 1,373 fecal samples distributed in 2,962 sampling hours, S2 and S3 Tables in S1 Appendix). Seeds belonged to 98 species (including seven alien tree species in small fragments) from eight growth forms (63 species of trees, 9 vines, 3 shrubs, palms, and lianas each, and 1 epiphyte, parasite, and herb each) distributed in 62 genera and 38 families (Table C in S1 Appendix). Howlers dispersed seeds of 30 to 53 species in small fragments and of 39 to 43 species in large ones. The actual values can be up to 37% higher according to nonparametric richness estimators (Table 1, see also Table C in S1 Appendix). The richness of dispersed seeds between small and large fragments often did not differ in pairwise comparisons (Fig 1). Group S3 was an exception as it dispersed lower seed richness than S1, S2, and L3, but it did not differ from L1 and L2 (Fig 1). Similarly, there was no difference between fragments in the number of dispersed seeds (χ^2^ = 9, df = 5, p = 0.1).

**Fig 2:**
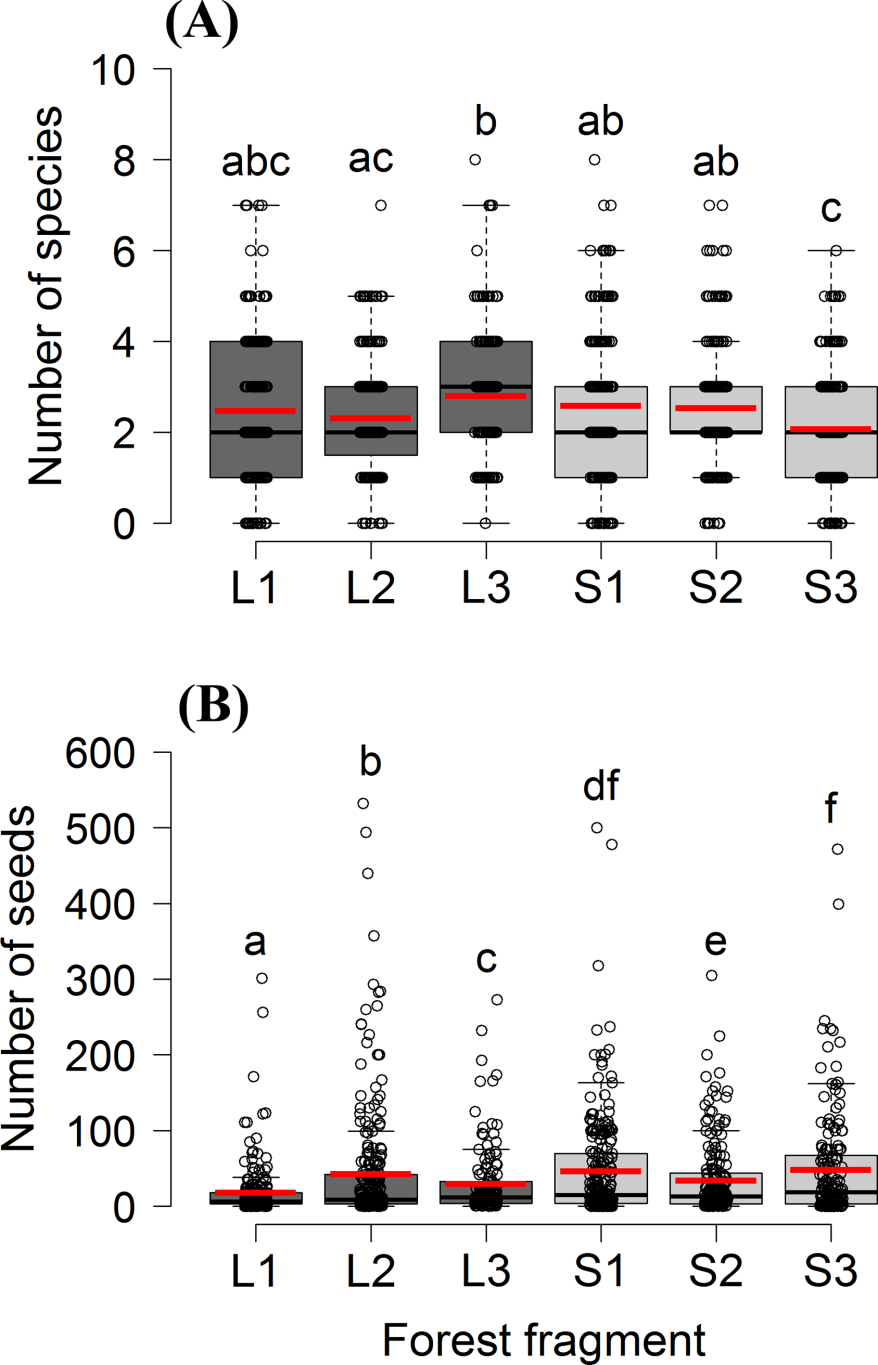
**Total richness of seed species (A) and the abundance of seeds >2 mm (B) at the fecal sample level.** The line within a box represents the median, the red line represents the mean, the box represents the range between the first and third interquartiles (IQR), and the whiskers represent the IQR multiplied by 1.5. Dots represent the fecal samples for each fragment. Boxes sharing no letter are significantly different (contrast tests, p<0.05).

The richness of seed species per fecal sample (range = 0–8; mean ± SD = 2 ± 1; n = 1,373 samples) differed among groups (GLM, F_(5,1365)_ = 6.5, p<0.0001; Fig 2A) because of a higher richness in L3 (mean ± SD = 3 ± 2) than in L2 and S3 (2 ± 1 both, contrast test, p<0.001; Fig 2A). The number of seeds >2 mm per sample (range = 0–532; median = 11) was higher in small fragments (GLM, F_(5,1150)_ = 6.4, p<0.0001, contrast test, p<0.001 in all cases; Fig 2B), where some fecal samples contained up to 500 seeds (mean ± SD = 47 ± 59) of the cultivated *Psidium guajava*. We estimated, based on the mean frequency of individual defecation events per day, that each brown howler dispersed an average of 143 (± 49) seeds >2 mm per day or 52,052 (± 17,782) of these seeds per year (S1 Table in S1 Appendix).

### Proportion of local tree assemblages dispersed by brown howlers

**Fig 3:**
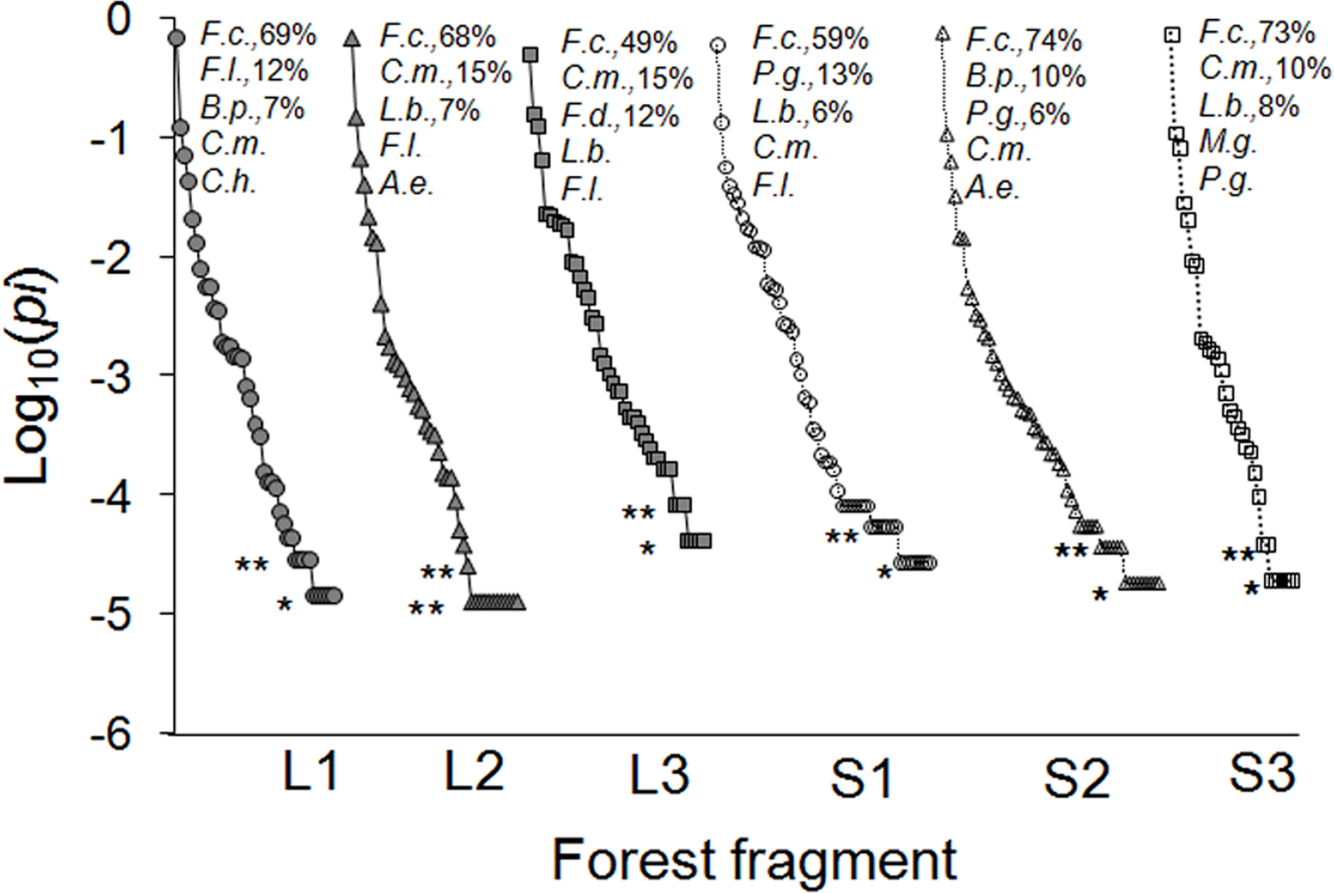
Rank-abundance graphs of seed species dispersed by brown howlers in each study fragment. The X-axis ranks species from the most to the least abundant. Species representing ~30% of all dispersed seeds in each fragment are indicated with two letters. Asterisks indicate species with only one (*singletons) or two (**doubletons) seeds. Underlined species dominate tree communities. A.e. = *Allophylus edulis* (Sapindaceae), B.p. = *Banara parviflora* (Salicaceae), C.h. = *Cereus hildmannianus* (Cactaceae), C.m. = *Coussapoa microcarpa* (Urticaceae), F.a. = *Ficus adhatodifolia* (Moraceae), F.c. = *F*. *cestrifolia*, F.l. = *F*. *luschnathiana*, P.g. = *Psidium guajava*(Myrtaceae), L.b. = *Lithraea brasiliensis* (Anacardiaceae), M.g. = *Myrcia glabra* (Myrtaceae). The abundance (proportion of the total number of seeds) of the three most prevalent species in each fragment is also shown.

The seed species richness dispersed by brown howlers represented between 46% and 79% of the total tree richness of each fragment, most of which (58%-93%) fit into the vertebrate seed-dispersal syndrome; that is, fleshy fruits (Table B in S1 Appendix). Nevertheless, 8 to 17 species accounted for most of the dispersed seeds in all study fragments (Table 2), particularly the fig tree *Ficus cestrifolia* (Table 2, Fig 3). This small-seeded species represented 49% to 74% of the total number of dispersed seeds in each fragment (Fig 3). Other top ranking species accounted for ≤15% of the dispersed seeds (Fig 3).

**Table 2:** Top species dispersed by brown howlers and percentage of damaged seeds in six Atlantic forest fragments.

Species	% of total fecal records (# fecal samples)						% damaged seeds (n)					
	S1	S2	S3	L1	L2	L3	S1	S2	S3	L1	L2	L3
*Ficus cestrifolia*	11.5 (68)	13.4 (58)	21.9 (69)	8.1 (34)	11.8 (59)	10.7 (40)	0 (1501)	0 (3486)	0 (3466)	0 (2947)	0 (2285)	0 (1841)
*Coussapoa microcarpa*	4.1 (24)	8.3 (36)	9.8 (31)	3.1 (13)	4.6 (23)	6.4 (24)	0 (515)	0 (1025)	0 (849)	0 (210)	0 (1237)	0 (1731)
*Lithraea brasiliensis*	6.4 (38)	2.5 (11)	15.9 (50)	―	9.8 (49)	5.6 (21)	0 (2047)	0 (160)	0 (4243)	―	0 (4521)	0 (1569)
*Guapira opposita*	3.1 (18)	2.3 (10)	―	3.6 (15)	7.6 (38)	3.7 (14)	0 (96)	0 (176)	―	0 (380)	0 (1024)	0 (218)
*Syagrus romanzoffiana*	8.1 (48)	―	―	12 (50)	13.4 (67)	6.4 (24)	3 (191)	―	―	0 (226)	0 (288)	5.1 (78)
*Diospyros inconstans*	5.6 (33)	―	―	4.5 (19)	6.4 (32)	10.2 (38)	2 (197)	―	―	0.1 (90)	0 (134)	0.9 (454)
*Banara parviflora*	―	5.3 (23)	2.9 (9)	9.6 (40)	―	2.7 (10)	―	0 (4714)	0 (480)	0 (4775)	―	0 (462)
*Enterolobium contortisiliquum*	4.8 (28)	2.3 (10)	―	―	7.4 (37)	4 (15)	4 (50)	6.3 (16)	―	―	7.5 (99)	17.8 (45)
*Ficus luschnathiana*	2.7 (16)	―	―	6.2 (26)	4 (20)	2.7 (10)	0.2 (928)	―	―	0 (8443)	0 (3173)	0 (559)
Psidium guajava*	14.1 (83)	13.2 (57)	16.2 (51)	―	―	―	0 (4842)	0 (2935)	0 (1061)	―	―	―
*Erythroxylum argentinum*	4.4 (26)	―	―	―	5 (25)	4 (15)	0 (419)	―	―	―	0 (1145)	0 (538)
*Allophylus edulis*	―	3 (13)	―	―	6.4 (32)	3.5 (13)	―	0 (783)	―	―	0 (1606)	0 (482)
*Myrcia glabra*	―	3.2 (14)	9.5 (30)	―	―	―	―	0 (242)	0 (1471)	―	―	―
*Annona sylvatica*	―	2.1 (9)	―	5.5 (23)	―	3.5 (13)	―	0 (32)	―	0 (114)	―	0 (31)
Hovenia dulcis*	2.7 (16)	6.7 (29)	―	―	―	―	1.6 (438)	3 (683)	―	―	―	―
*Trichilia claussenii*	―	―	―	4.5 (19)	3.8 (19)	―	―	―	―	0 (75)	0 (87)	―
Citrus reticulata*	4.6 (27)	3.5 (15)	―	―	―	―	0 (591)	0 (47)	―	―	―	―
Syzygium cummini*	―	4.8 (21)	3.2 (10)	―	―	―	―	0 (36)	1.7 (59)	―	―	―
*Campomanesia xanthocarpa*	3.2 (19)	―	2.9 (9)	―	―	―	―	―	0.9 (109)	―	―	―
*Cecropia pachystachya*	3.4 (20)	―	―	―	―	2.4 (9)	0 (1210)	―	―	―	―	0 (409)
*Cordia ecalyculata*	―	―	―	6 (25)	―	―	―	―	―	0 (77)	―	―
*Hyperbaena domingensis*	―	―	―	―	―	5.9 (22)	―	―	―	―	―	0 (163)
*Garcinia gardneriana*	―	―	―	5.5 (23)	―	―	―	―	―	0 (253)	―	―
Eriobotrya japonica*	―	5.1 (22)	―	―	―	―	―	0 (98)	―	―	―	―
*Cereus hildmannianus*	―	―	―	6.7 (28)	―	―	―	―	―	0 (1416)	―	―
*Myrcianthes pungens*	―	―	―	3.8 (16)	―	―	―	―	―	0.2 (452)	―	―
*Chrysophyllum marginatum*	3.7 (22)	―	―	―	―	―	2 (98)	―	―	―	―	―
*Ficus adhatodifolia*	―	―	―	―	―	3.7 (14)	―	―	―	―	―	0 (2026)
*Eugenia rostrifolia*	―	―	―	3.6 (15)	―	―	―	―	―	0 (90)	―	―
*Myrciaria cuspidata*	―	3.5 (15)	―	―	―	―	―	0 (120)	―	―	―	―
*Chrysophyllum gonocarpum*	―	3 (13)	―	―	―	―	―	0 (69)	―	―	―	―
*Celtis iguanaea*	―	―	―	―	―	2.7 (10)	―	―	―	―	―	0 (13)
*Prunus myrtifolia*	―	―	―	―	―	2.4 (9)	―	―	―	―	―	0 (66)
	Total species						Mean (SD) of the % of damaged seeds					
	**15**	**16**	**8**	**14**	**11**	**17**	**1.1 (1.3)**	**0.6 (1.7)**	**0.4 (0.6)**	**0.1 (0.3)**	**0.7 (2.3)**	**1.4 (4.4)**

* Cultivated alien species (n = 5). Seed species (n = 33) organized in decreasing order of the number of fragments where they were defecated and then the percentage of total fecal records. See also S3 Table for a complete list of dispersed seed species.

### Diversity of dispersed seeds

**Fig 4:**
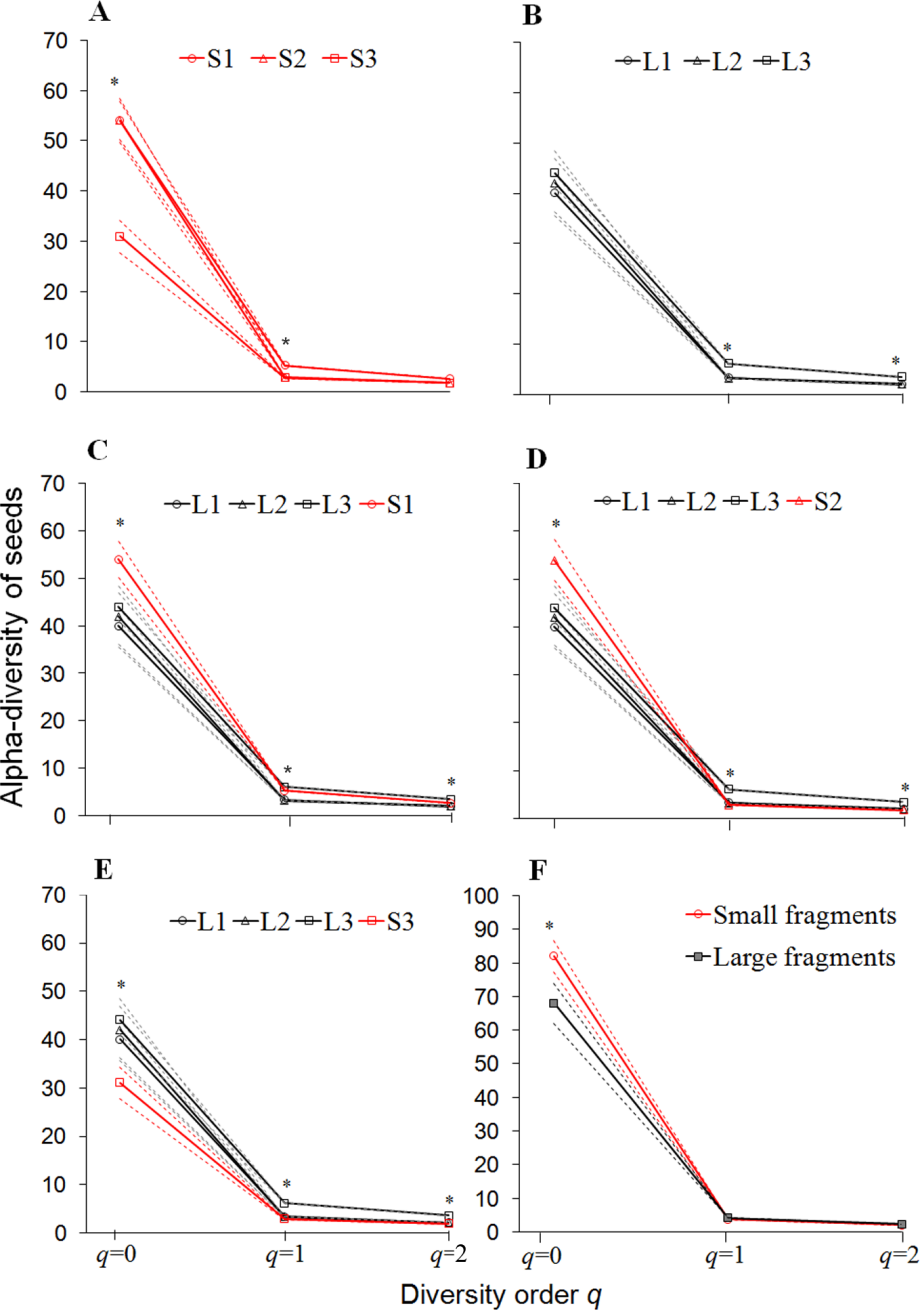
Alpha-diversity of seeds dispersed by brown howlers in six Atlantic forest fragments in southern Brazil. Diversity comparisons between small fragments (A), large fragments (B), large fragments and each small fragment (C-E), and pooled seed diversity for both habitat types (F). The 95% confidence intervals (CI) for each small fragment are highlighted with red dotted lines. Non-overlapping 95% CIs indicate significant differences in species richness (*q* = 0), and the diversity of common (*q* = 1) and dominant (*q* = 2) species. Significant differences (p<0.05) indicated with asterisks.

We found differences in the alpha-diversity of seed species dispersed within both small and large fragments (Fig 4A and 4B). Overall, howlers inhabiting the small fragments S1 and S2 dispersed seeds of a higher diversity of rarely eaten fruit species (order *q* = 0) and/or common species than those inhabiting S3 (Fig 4A). Among large fragments, the diversity of common (*q* = 1) and dominant (*q* = 2) species was higher in L3 than in L1 and L2 (Fig 4B). The diversity of rare seed species was higher in small, except S3, than in large fragments (Fig 4C–4E). On the other hand, there was no clear pattern in the diversity of common and dominant species, except for an overall higher diversity in L3 compared with all the other fragments (Fig 4C–4E). Finally, the overall diversity of rare seed species at the forest level was higher in small than in large fragments (Fig 4F).

### Qualitative components of seed dispersal

**Fig 5:**
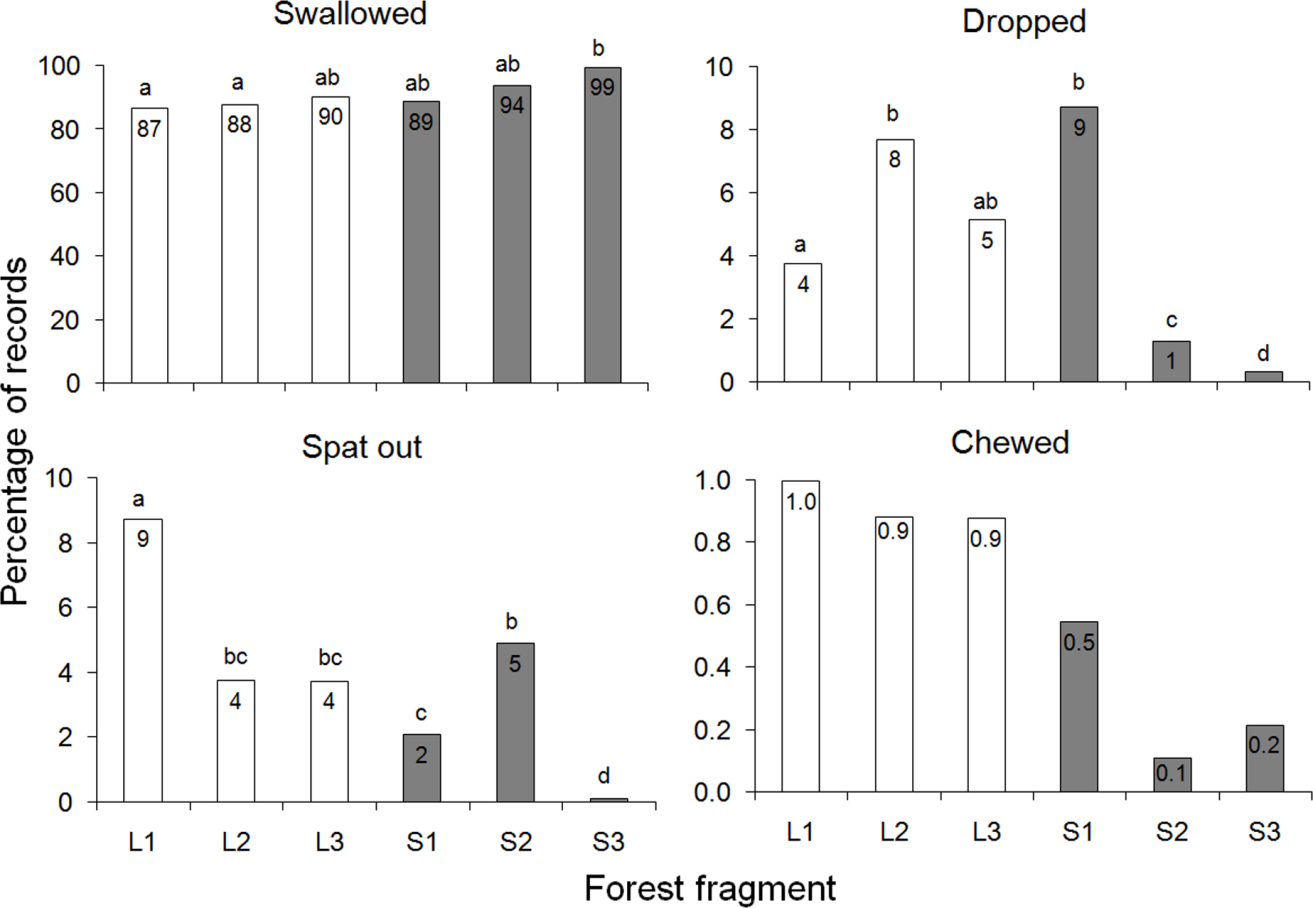
Patterns of seed handling by brown howlers in six Atlantic forest fragments in southern Brazil. We used rarefied data to calculate the proportion of records devoted to each seed handling type (n = 937 records per group). The numbers within the bars indicate the percentage. Different letters above bars indicate significant between-fragment differences (p<0.05). Observe that the scales in the Y-axes differ.

Howlers swallowed most seeds in both small and large fragments (mean ± SD = 91% ± 5%, range = 87%-99%), and they dropped or spat out a similar or lower amount of seeds in small than in large fragments (Supporting information S1 Appendix, Fig 5). However, the proportion of chewed seeds was similarly low (range = 0.1–1%) in all groups (p>0.05 in all contrasts, Fig 5). Howlers defecated intact most swallowed seeds in all fragments (range = 96%-~100%; GLM, F_(5,245)_ = 2.6, p = 0.1, Figure A in S1 Appendix). Overall, only 0.1% (or 132 out of 92,243 seeds) of dispersed seeds had moderate, medium or heavy testa damage. The damaged seeds belonged to 19 species, particularly *Enterolobium contortisiliquum* (4%-18% of the seeds defecated by different groups, seed size = 16 mm), *Hovenia dulcis* (2%-3%, size = 6 mm), and *Chrysophyllum marginatum* (2%, size = 10 mm, Table 2, Table C in S1 Appendix).

## Discussion

Overall, we found support for our prediction that brown howlers provide a striking seed dispersal service to the tree species of the Atlantic forest fragments that they inhabit in southern Brazil, thereby potentially contributing significantly to the maintenance of their community structure. We observed that they are legitimate seed dispersers of 98 plant species that together represented most of the vertebrate-dispersed species of each fragment. Although secondary seed dispersers, seed predators, and pathogens can modify seed fate [45, 46] and the pattern of seed deposition produced by primary dispersers [4–6], the transport away from the parent tree and the removal of pulp/aril germination inhibitors are likely to be significantly reduced in the absence of primates [18, 19, 35, 46].

The richness of seeds dispersed by each study group was 10% to 380% higher than previously reported for brown howler groups (14 spp., [47]; 23–27 spp., [48]), but 50% to 450% lower than reported for Amazonian *A*. *seniculus* groups (80–137 spp., [35]). It was also near the upper limit recorded in other howler monkeys (range = 5–39 spp., reviewed by [35]). Overall, the number of seed species dispersed by brown howlers is even higher than the number previously reported for the genus *Alouatta* in the Atlantic forest (74 spp., [18]), a discrepancy explained by the factors described below.

It is likely that the observed patterns of richness of dispersed seeds are more related to methodological (e.g. sampling effort: [35]) and between-site differences in the composition of plant assemblages among studies than to an ‘actual’ gradient of seed dispersal effectiveness among species. Overall, there is a positive relationship between sampling effort and the richness of dispersed seeds [35] because the length of the fruiting season varies between species from a few weeks to many months per year or even in two or more year-cycles [49]. The predominance of studies lasting <12 months in the literature undoubtedly underestimates our ability to assess the role of frugivores, including howler monkeys, in forest structuring [35]. Therefore, we highlight the importance of multiyear field studies to better estimate the richness of seeds that a frugivorous species disperses.

As predicted, we did not find evidence that brown howlers disperse a lower number of seeds belonging to a poorer tree assemblage at the forest and fecal sample levels in small fragments in comparison with larger ones. Conversely, we found support for the idea that howlers compensate the potential reduction in preferred native fruit sources in small fragments by exploiting second-choice native fruit species or cultivated alien species that grow at the fragment’s edge or the nearby matrix [25, 28, 30, 50]. This diet supplementation with cultivated species also explains why the number of dispersed seeds >2 mm (e.g. *P*. *guajava*) was higher in small than in large fragments.

To the best of our knowledge, this is the first study showing that a single primate species provides seed dispersal services for most of the vertebrate-dispersed tree species of the habitat patches that it inhabits. Given that we monitored each study group once every two months, it is likely that even a higher proportion of the tree species from this syndrome that are found in their forest fragments benefit from the seed dispersal promoted by the eclectic fruit-feeding howlers [27, 28, 31]. The fact that howler monkeys are the last large remnant arboreal frugivores of the original fauna [51] that are capable of ingesting and dispersing large seeds (at least, up to 23 mm as those of “bacupari” *Garcinia gardneriana*) in this region further highlights the importance of these dispersers in the region.

In addition to the dispersal of large seeds, howlers are important dispersers for small-seeded species. This is the case for figs (*Ficus* spp.). Fig trees are important keystone food sources for *Alouatta* spp. [25, 35, 52], including brown howlers [27], as well as for many frugivorous birds (990 species) and terrestrial mammals (284 species) [53]. For instance, seeds of a single fig species (*F*. *cestrifolia*: ≤1 mm, fruits ~15 mm in diameter) widespread in most forest remnants of southern Atlantic forest [36] dominated the assemblage of dispersed seeds of all study groups. The characteristic asynchronous fruit phenology and high nutritional value of *Ficus* [53, 54] elevate its importance to the diet of brown howlers.

We found that the alpha-diversity of seed species, particularly rare species, was often similar or higher in small than in large fragments. This finding challenges the presumably lower food availability for atelids in small or low-quality habitats (e.g. [17, 30, 35]). Indeed, despite the tenfold difference in size between small and large fragments, our six forest fragments varied only slightly in tree species richness (Table B in S1 Appendix). The importance of top food species (and, probably, their absolute density), however, was lower in small than in large fragments [27]. Here again the howler dietary flexibility [25, 26–28] helps to explain the higher richness of seeds from rarely eaten fruits dispersed in small fragments.

The role of brown howlers as facilitators of forest regeneration is particularly critical for the maintenance of the structure of the plant assemblage of small Atlantic forest fragments because their guilds of seed dispersers are further impoverished [10]. As recently shown for Amazonian forest fragments [11], the conservation of small forest remnants in the heavily fragmented Atlantic forest [21] is crucial for the long-term persistence of its forested ecosystems because of their role as reservoirs of plant propagules. The contribution of small forest fragments to viable metapopulations of seed dispersers >5 kg (such as brown howlers) also adds to their importance in the maintenance and recovery of the original floristic diversity.

Finally, brown howlers are also qualitatively efficient legitimate seed dispersers because they ingest most seeds of the fruits that they eat and they defecate them intact. This kind of seed handling that favors the survival, germination, and recruitment of dispersed seeds [2, 4, 6, 7, 19] was the norm in both small and large fragments. In this respect, howlers can qualify as more efficient seed dispersers than those highly frugivorous atelids that damage or drop higher proportions of seeds under parent trees, particularly in small fragments (e.g. *Ateles geoffroyi*, [17]). Germination rates of >1-cm seeds of five species ingested by *Alouatta pigra* and *A*. *geoffroyi* support that howlers provide higher quality dispersal than do spider monkeys [55]. Studies comparing the patterns of seed handling, damage level, and germination of seeds dispersed by brown howlers and other Atlantic forest arboreal frugivores (e.g. *Brachyteles hypoxanthus*, *Sapajus* spp., and *Ramphastos* spp.) are necessary to assess the putative advantage of howler monkeys in providing this ecosystem service.

In sum, we highlight the importance of multiyear research in evaluating the quantitative and qualitative role that frugivorous species play as efficient seed dispersers. We showed that a single frugivorous species can provide efficient seed dispersal services for most tree species of the communities of severely disturbed and defaunated Atlantic forest fragments [10, 21]. Therefore, the loss of brown howlers and other large arboreal Atlantic forest frugivores can compromise seed dispersal and seedling recruitment, ultimately changing the composition of plant communities as has been recorded in other tropical regions (e.g. [15, 16]). Future studies should compare the fate of seeds dispersed by the members of the guilds of primary and secondary dispersers, the impact of seed predators on seedling recruitment, the dispersal distances, the seed shadows created by different frugivores, and the impact of population declines of particular frugivorous species on seed dispersal services between forest patches that vary in size and disturbance level. This knowledge is critical for our understanding of the role played by frugivorous species on the structuring of plant communities and for informing conservation management plans in imperiled biomes, such as the Atlantic forest.

## Supporting information

S1 Appendix Supplemental results on seed handling. Table A. Sampling effort and numbers of seeds dispersed by brown howlers.
Table B. Tree species richness and percentage of tree species dispersed.
Table C. Seed species dispersed by brown howlers.
Figure A. Mean proportion of seeds defecated intact by brown howlers in large and small fragments.
(DOC)
