# Supplementary material for: Quantity and quality of seed dispersal by a large arboreal frugivore in small and large Atlantic forest fragments
Source: PLoS One. 2018 Mar 21;13(3):e0193660. doi: 10.1371/journal.pone.0193660 (PMC5862440; doi:10.1371/journal.pone.0193660)
Supplement: S1 Appendix — Table A. Sampling effort and numbers of seeds dispersed by brown howlers. Table B. Tree species richness and percentage of tree species dispersed. Table C. Seed species dispersed by brown howlers. Figure A. Mean proportion of seeds defecated intact by brown howlers in large and small fragments. (DOC) [file pone.0193660.s001.doc]

**Supporting information**

Chaves ÓM, Bicca-Marques JC, Chapman CA. 2007. Quantity and quality of seed dispersal by a large arboreal frugivore in small and large Atlantic forest fragments. PlosOne

**S1 Appendix.** Supplemental results on seed handling

Between-group differences were found in the proportion of seeds swallowed (χ^2^ = 17.6; d.f.=5, p = 0.003), dropped (χ^2^ = 115; d.f.=5, p < 0.0001), and spat out (χ^2^ = 98; d.f.=5, p < 0.0001), while the percentage of chewed seeds was negligible and did not differ among groups (χ^2^ = 10; d.f.=5, p = 0.06; S3 Fig). Overall, group S3 dropped and spat out less seeds than the other groups (S3 Fig).

**Table A**. Sampling effort and number of seeds dispersed by brown howlers in six Atlantic forest fragments in southern Brazil.

| Fragment | Group size^a^ | Sampling effort | | | | | | %EF^d^ | TDS^e^ at group level | |  | TDS at individual level | |
| --- | --- | --- | --- | --- | --- | --- | --- | --- | --- | --- | --- | --- | --- |
|  |  | Years | Months | Days | Hours | FS^b^ | # seeds^c^ |  | Day | Year |  | Day | Year |
| S1 | 6 (6) | 4 | 29 | 62 | 501 | 260 | 36,879 | 6.5 | 1,122 | 408,408 |  | 187 | 68,068 |
| S2 | 10 (9) | 4 | 27 | 58 | 446 | 224 | 54,257 | 3.1 | 1,101 | 400,764 |  | 122 | 44,529 |
| S3 | 9 (8) | 4 | 25 | 65 | 550 | 218 | 51,981 | 6.9 | 1,557 | 566,748 |  | 195 | 70,844 |
| L1 | 10 (8) | 4 | 23 | 77 | 486 | 226 | 68,285 | 9.3 | 592 | 215,306 |  | 74 | 26,913 |
| L2 | 9 (8) | 4 | 24 | 81 | 550 | 288 | 79,519 | 3.5 | 1,380 | 502,320 |  | 173 | 62,790 |
| L3 | 9 (9) | 4 | 22 | 63 | 429 | 157 | 24,692 | 5.2 | 967 | 351,988 |  | 107 | 39,110 |
|  | **53 (48)** | **4** | **36** | **291** | **2962** | **1373** | **315,613** | **5.7** (**2)** | **1,120** (**335)** | **407,589 (121,997)** |  | **143 (49)** | **52,052 (17,782)** |
|  |  | Total | | | | | | mean (± SD) | | | | | |

^a^ The number of individuals excluding infants is indicated in parentheses.

^b^ Number of analyzed fresh fecal samples (FS) containing at least one seed species.

^c^ Estimated number of seeds found in all fecal samples. All seeds >2 mm were counted. The number of smaller species (e.g., *Ficus* spp. and *Banara parviflora*) was estimated based on abundance categories when the fecal sample contained >100 seeds (see Methods).

^d^ Percentage of fecal samples containing no seed.

^e^ Total number of dispersed seeds (TDS). TDS at the group level per day was estimated based on the following formula: mean number of seeds >2 mm per sample x number of non-infant individuals in the group x mean number of defecation events per day (=4). TDS at the individual level was estimated based on the following formula: TDS at the group level per day/number of non-infant individuals in the group.

**Table B.** Tree species richness and percentage of tree species dispersed by howler monkeys in six Atlantic forest fragments in southern Brazil.

| Fragment | Tree richness^a^ | |  | # dispersed tree species | | |
| --- | --- | --- | --- | --- | --- | --- |
|  | Total | VD-species^b^ |  | Total | %TTR^c^ | %VD-species |
| S1 | 61 | 50 |  | 39 | 63.9 | 78.0 |
| S2 | 48 | 41 |  | 38 | 79.2 | 92.7 |
| S3 | 54 | 43 |  | 25 | 46.3 | 58.1 |
| L1 | 48 | 38 |  | 30 | 62.5 | 78.9 |
| L2 | 51 | 44 |  | 30 | 58.8 | 68.2 |
| L3 | 51 | 39 |  | 34 | 66.7 | 87.2 |

^a^ Shrubs and palms are also included here. Full details of tree surveys are provided in Chaves and Bicca-Marques (2016).

^b^ Vertebrate-dispersed tree species (i.e. those producing diaspores attached to fleshy pulp or aril). The remaining species corresponded to abiotic-dispersed species (i.e. species producing dry winged seeds or fruits).

^c^ Percentage of total tree richness.

**Table C. Seed species dispersed by brown howlers in six Atlantic forest fragments in southern Brazil.**

| Species | Family | G.F.^a^ | Size^b^ | % of total fecal records | | | | | |
| --- | --- | --- | --- | --- | --- | --- | --- | --- | --- |
|  |  |  |  | S1 | S2 | S3 | L1 | L2 | L3 |
| *Ficus cestrifolia* | Moraceae | Tree | 1 | 11.6 | 13.5 | 21.9 | 8.1 | 11.8 | 10.7 |
| *Syagrus romanzoffiana* | Arecaceae | Palm | 19 | 8.2 | 4.9 | 2.9 | 12.0 | 13.4 | 6.4 |
| *Lithraea brasiliensis* | Anacardiaceae | Tree | 6 | 6.5 | 2.6 | 15.9 | 2.9 | 9.8 | 5.6 |
| *Coussapoa microcarpa* | Urticaceae | Tree | 2.5 | 4.1 | 8.4 | 9.8 | 3.1 | 4.6 | 6.4 |
| *Diospyros inconstans* | Ebenaceae | Tree | 12 | 5.6 | 1.2 | 0.3 | 4.5 | 6.4 | 10.2 |
| *Myrcia glabra* | Myrtaceae | Tree | 8 | 2.2 | 3.2 | 9.5 | 0.2 | 0.8 | 0.5 |
| *Campomanesia xanthocarpa* | Myrtaceae | Tree | 6 | 3.2 | 0.7 | 2.9 | 1.9 | 0.2 | 0.8 |
| *Banara parviflora* | Salicaceae | Tree | 1 | 2.2 | 5.3 | 2.9 | 9.6 | ― | 2.7 |
| *Guapira opposita* | Nyctaginaceae | Tree | 11 | 3.1 | 2.3 | ― | 3.6 | 7.6 | 3.8 |
| *Enterolobium contortisiliquum* | Fabaceae | Tree | 16 | 4.8 | 2.3 | 0.6 | ― | 7.4 | 4.0 |
| *Allophylus edulis* | Sapindaceae | Tree | 7 | 2.4 | 3.0 | ― | 2.9 | 6.4 | 3.5 |
| *Erythroxylum argentinum* | Erythroxylaceae | Tree | 10 | 4.4 | 0.5 | ― | 0.2 | 5.0 | 4.0 |
| *Annona sylvatica* | Annonaceae | Tree | 14 | 1.0 | 2.1 | ― | 5.5 | 2.0 | 3.5 |
| *Hyperbaena domingensis* | Menispermaceae | Vine | 16 | 0.9 | 1.4 | ― | 0.2 | 0.8 | 5.9 |
| *Chrysophyllum marginatum* | Sapotaceae | Tree | 10 | 3.7 | 0.7 | ― | 1.2 | 2.2 | 0.3 |
| *Casearia decandra* | Salicaceae | Tree | 1 | 0.5 | ― | 2.2 | 0.7 | 0.6 | 2.1 |
| *Myrsine umbellata* | Primulaceae | Tree | 6 | 1.2 | 0.9 | ― | 0.2 | 1.4 | 1.9 |
| *Myrceugenia glaucescens* | Myrtaceae | Tree | 10 | 0.2 | 0.2 | ― | 0.5 | 0.2 | 0.5 |
| *Psidium guajava** | Myrtaceae | Tree | 3.5 | 14.1 | 13.2 | 16.2 | ― | ― | 1.6 |
| *Ficus luschnathiana* | Moraceae | Tree | 2 | 2.7 | ― | ― | 6.2 | 4.0 | 2.7 |
| *Trichilia claussenii* | Meliaceae | Tree | 21 | ― | 0.7 | ― | 4.5 | 3.8 | 0.5 |
| *Eugenia rostrifolia* | Myrtaceae | Tree | 9 | 0.2 | 1.6 | ― | 3.6 | 0.6 | ― |
| *Myrciaria cuspidata* | Myrtaceae | Tree | 8 | 0.3 | 3.5 | 1.3 | ― | 0.8 | ― |
| *Schinus terebinthifolius* | Anacardiaceae | Tree | 6 | 0.2 | 0.9 | 2.2 | ― | ― | 0.3 |
| *Celtis iguanaea* | Cannabaceae | Liana | 9 | 0.2 | 0.2 | ― | ― | 0.2 | 2.7 |
| *Passiflora elegans* | Passifloraceae | Vine | 4 | 0.2 | ― | ― | 0.2 | 1.0 | 0.3 |
| Msp. 5 | UNK | UNK | 10 | ― | 0.2 | 0.3 | 0.2 | 0.2 | ― |
| *Hovenia dulcis** | Rhamnaceae | Tree | 6 | 2.7 | 6.7 | 0.6 | ― | ― | ― |
| *Citrus reticulata** | Rutaceae | Tree | 12 | 4.6 | 3.5 | ― | ― | ― | 0.5 |
| *Cordia ecalyculata* | Boraginaceae | Tree | 9 | ― | ― | 0.3 | 6.0 | 2.2 | ― |
| *Cecropia pachystachya* | Urticaceae | Tree | 1 | 3.6 | 0.2 | ― | ― | ― | 2.4 |
| *Chrysophyllum gonocarpum* | Sapotaceae | Tree | 15 | 0.3 | 3.0 | ― | ― | ― | 1.6 |
| *Psidium cattleianum* | Myrtaceae | Tree | 3.3 | ― | ― | 1.3 | ― | 0.8 | 1.3 |
| *Sorocea bonplandii* | Moraceae | Tree | 12 | 0.2 | ― | ― | ― | 1.0 | 1.3 |
| *Nectandra megapotamica* | Lauraceae | Tree | 9 | ― | 0.7 | 0.6 | 0.7 | ― | ― |
| *Vitex megapotamica* | Lamiaceae | Tree | 10 | ― | ― | 0.3 | 1.4 | 0.2 | ― |
| *Sebastiania serrata* | Euphorbiaceae | Tree | 4 | 0.3 | 0.5 | ― | 0.5 | ― | ― |
| *Phoradendron* c.f. *argentinum* | Santalaceae | Parasite | 3 | ― | 0.2 | ― | 0.2 | ― | 0.8 |
| Msp. 2 | UNK | UNK | 4 | 0.2 | 0.7 | 0.3 | ― | ― | ― |
| Msp. 6 | UNK | UNK | 3 | ― | 0.2 | 0.3 | ― | ― | 0.5 |
| *Passiflora* Msp. 1 | Passifloraceae | Vine | 4 | 0.2 | ― | ― | 0.2 | 0.2 | ― |
| *Cereus hildmannianus* | Cactaceae | Tree | <1 | ― | ― | ― | 6.7 | 0.4 | ― |
| *Garcinia gardneriana* | Clusiaceae | Tree | 23 | 0.2 | ― | ― | 5.5 | ― | ― |
| *Eriobotrya japonica** | Rosaceae | Tree | 20 | ― | 5.1 | 0.3 | ― | ― | ― |
| *Ficus adhatodifolia* | Moraceae | Tree | 2.5 | ― | ― | 0.3 | ― | ― | 3.8 |
| *Myrcianthes pungens* | Myrtaceae | Tree | 11 | ― | ― | ― | 3.8 | 0.2 | ― |
| *Eugenia* sp.2 | Myrtaceae | Tree | 6 | ― | 0.2 | 1.6 | ― | ― | ― |
| *Faramea montevidensis* | Rubiaceae | Tree | 8 | ― | 0.2 | ― | 1.0 | ― | ― |
| *Eugenia* sp. 1 | Myrtaceae | Tree | 8 | ― | 0.5 | 0.6 | ― | ― | ― |
| *Zanthoxylum* sp. 1 | Rutaceae | Tree | 2 | ― | ― | 0.3 | ― | ― | 0.5 |
| *Butia capitatta* | Arecaceae | Palm | 15 | ― | ― | ― | 0.2 | 0.6 | ― |
| Msp. 8 | UNK | UNK | 7 | ― | 0.5 | ― | ― | ― | 0.3 |
| *Araucaria angustifolia** | Araucariaceae | Tree | 51 | 0.3 | ― | ― | ― | ― | 0.3 |
| Msp. 12 | UNK | UNK | <1 | ― | ― | ― | 0.2 | ― | 0.3 |
| Msp. 11 | UNK | UNK | 3 | ― | 0.2 | ― | ― | 0.2 | 0.0 |
| *Myrsine coriacea* | Primulaceae | Tree | 6 | ― | 0.2 | ― | ― | 0.2 | 0.0 |
| *Passiflora foetida* | Passifloraceae | Vine | 5 | 0.2 | ― | ― | 0.2 | ― | ― |
| Msp. 7 | UNK | UNK | 4 | 0.2 | 0.2 | ― | ― | ― | ― |
| *Syzygium cummini** | Myrtaceae | Tree | 22 | ― | ― | 3.2 | ― | ― | ― |
| *Prunus myrtifolia* | Rosaceae | Tree | 10 | ― | ― | ― | ― | ― | 2.4 |
| *Zanthoxylum fagara* | Rutaceae | Tree | 3 | ― | ― | ― | ― | 2.2 | ― |
| *Chomelia obtusa* | Rubiaceae | Shrub | 10 | ― | ― | ― | ― | ― | 1.3 |
| Msp. 10 | UNK | UNK | 3 | ― | 0.7 | ― | ― | ― | ― |
| *Eugenia* sp.3 | Myrtaceae | Tree | 7 | ― | ― | 0.6 | ― | ― | ― |
| *Cupania vernalis* | Sapindaceae | Tree | 11 | ― | ― | ― | 0.5 | ― | ― |
| *Eugenia uniflora* | Myrtaceae | Tree | 8 | ― | 0.5 | ― | ― | ― | ― |
| *Merremia dissecta* | Convolvulaceae | Vine | 11 | ― | ― | ― | ― | ― | 0.5 |
| Msp. 4 | UNK | UNK | 5 | ― | 0.5 | ― | ― | ― | ― |
| *Pisonia aculeata* | Nyctaginaceae | Liana | 6 | 0.5 | ― | ― | ― | ― | ― |
| *Eugenia involucrata* | Myrtaceae | Tree | 10 | ― | ― | ― | ― | ― | 0.3 |
| *Heteropterys* sp. | Malpighiaceae | Vine | 9 | 0.3 | ― | ― | ― | ― | ― |
| *Inga striata* | Fabaceae | Tree | 14 | 0.3 | ― | ― | ― | ― | ― |
| Msp. 3 | UNK | UNK | 6 | 0.3 | ― | ― | ― | ― | ― |
| palm Msp. 1 | Arecaceae | Palm | 14 | 0.3 | ― | ― | ― | ― | ― |
| Poaceae Msp. 1 | Poaceae | Herb | 8 | ― | ― | ― | ― | ― | 0.3 |
| *Psychotria carthagenensis* | Rubiaceae | Shrub | 5 | ― | ― | ― | ― | ― | 0.3 |
| *Rhipsalis* c.f. *baccifera* | Cactaceae | Epiphyte | <1 | ― | ― | 0.3 | ― | ― | ― |
| vine Msp. 1 | UNK | Vine | 5 | 0.3 | ― | ― | ― | ― | ― |
| *Byrsonima ligustrifolia* | Malpighiaceae | Tree | 8 | ― | 0.2 | ― | ― | ― | ― |
| *Ceiba speciosa* | Malvaceae | Tree | 5 | 0.2 | ― | ― | ― | ― | ― |
| *Chiococca alba* | Rubiaceae | Shrub | 2 | 0.2 | ― | ― | ― | ― | ― |
| *Citharexylum myrianthum* | Verbenaceae | Tree | 9 | 0.2 | ― | ― | ― | ― | ― |
| *Cordia americana* | Boraginaceae | Tree | 11 | ― | ― | ― | 0.2 | ― | ― |
| *Eugenia pyriformis* | Myrtaceae | Tree | 8 | ― | 0.2 | ― | ― | ― | ― |
| *Inga vera* | Fabaceae | Tree | 16 | ― | 0.2 | ― | ― | ― | ― |
| *Ligustrum lucidum** | Oleaceae | Tree | 6 | ― | 0.2 | ― | ― | ― | ― |
| *Matayba elaeagnoides* | Sapindaceae | Tree | 13 | ― | ― | ― | ― | 0.2 | ― |
| Msp. 1 | UNK | UNK | 5 | ― | ― | ― | 0.2 | ― | ― |
| Msp. 9 | UNK | UNK | 6 | ― | 0.2 | ― | ― | ― | ― |
| Msp. 13 | UNK | UNK | <1 | 0.2 | ― | ― | ― | ― | ― |
| Msp. 14 | UNK | UNK | 8 | ― | ― | ― | ― | 0.2 | ― |
| *Passiflora* c.f. *suberosa* | Passifloraceae | Vine | 2.5 | 0.2 | ― | ― | ― | ― | ― |
| *Passiflora* Msp. 2 | Passifloraceae | Vine | 3 | ― | 0.2 | ― | ― | ― | ― |
| *Solanum pseudoquina* | Solanaceae | Tree | 4 | 0.2 | ― | ― | ― | ― | ― |
| *Solanum* sp. 2 | Solanaceae | Tree | 2 | 0.2 | ― | ― | ― | ― | ― |
| *Strychnos brasiliensis* | Loganiaceae | Liana | 12 | ― | ― | ― | ― | 0.2 | ― |
| *Styrax leprosum* | Styracaceae | Tree | 11 | 0.2 | ― | ― | ― | ― | ― |
| *Trema micrantha* | Cannabaceae | Tree | 6 | ― | 0.2 | ― | ― | ― | ― |
| Σ species (genera) | Σ families | Σ G.F. |  | Total fecal records | | | | | |
| **98 (62)** | **38** | **8** |  | **588** | **431** | **315** | **418** | **500** | **373** |

^a^ Growth form.

^b^ Mean seed size (mm) on the longer axis calculated based on 4 to 16 seeds.

UNK = Unknown.

*Alien species.


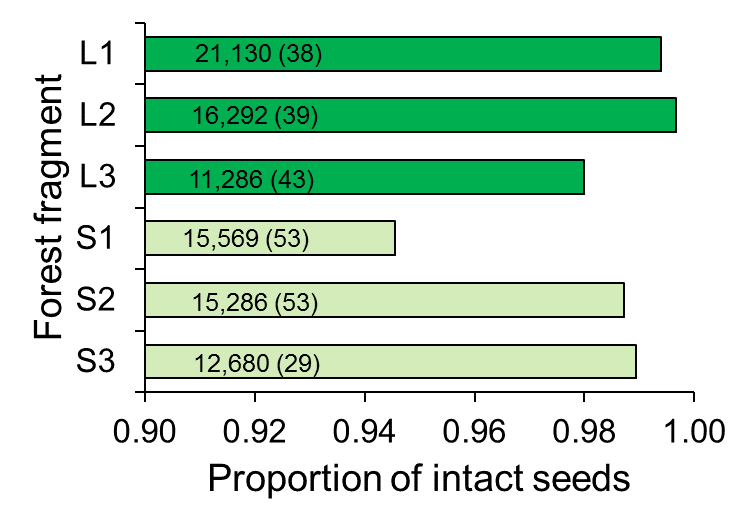


**Figure A.** Mean proportion of seeds defecated intact (undamaged) by brown howler monkeys in large (green bars) and small (olive bars) fragments. The number of analyzed seeds and the richness of seed species (in parentheses) are shown within the bars.
